# Recent Advancement in Optical Metasurface: Fundament to Application

**DOI:** 10.3390/mi13071025

**Published:** 2022-06-28

**Authors:** Naqeeb Ullah, Ruizhe Zhao, Lingling Huang

**Affiliations:** 1 Beijing Engineering Research Center of Mixed Reality and Advanced Display, School of Optics and Photonics, Beijing Institute of Technology, Beijing 100081, China; naqeeb.ullah@buitms.edu.pk (N.U.); zhaoruizhe@bit.edu.cn (R.Z.); 2 Department of Electronic Engineering, Balochistan University of Information Technology, Engineering and Management Sciences, Quetta 87300, Pakistan

**Keywords:** metasurfaces, cascaded meta-systems, tunable metasurfaces, vortex beams, holography

## Abstract

Metasurfaces have gained growing interest in recent years due to their simplicity in manufacturing and lower insertion losses. Meanwhile, they can provide unprecedented control over the spatial distribution of transmitted and reflected optical fields in a compact form. The metasurfaces are a kind of planar array of resonant subwavelength components that, depending on the intended optical wavefronts to be sculpted, can be strictly periodic or quasi-periodic, or even aperiodic. For instance, gradient metasurfaces, a subtype of metasurfaces, are designed to exhibit spatially changing optical responses, which result in spatially varying amplitudes of scattered fields and the associated polarization of these fields. This paper starts off by presenting concepts of anomalous reflection and refraction, followed by a brief discussion on the Pancharatanm–Berry Phase (PB) and Huygens’ metasurfaces. As an introduction to wavefront manipulation, we next present their key applications. These include planar metalens, cascaded meta-systems, tunable metasurfaces, spectrometer retroreflectors, vortex beams, and holography. The review concludes with a summary, preceded by a perspective outlining our expectations for potential future research work and applications.

## 1. Introduction

Metasurfaces are artificially engineered planar nanostructures composed of subwavelength resonant meta-atoms and have gained great attention owing to their abilities to reshape the optical nanoscale properties. For many years, researchers have exploited the inherent features of naturally occurring materials to construct numerous optical devices, including lenses, for example, that enable wavefront control by collecting phase and amplitude variations when the material is illuminated by the light. As a result, the refractive index of materials and the macroscopic structures of optical devices confine those typical optical components. The innovative two-dimensional metamaterials, also called metasurfaces, take a different approach to tailor light beams on the basis of abrupt and designable light modulation by ultrathin layer components [1,2]. Optical metasurfaces, considered two-dimensional metamaterial layers with promising features of spatially varying structure, demonstrate exceptional versatilities in altering the electromagnetic properties of the light within an optically narrow surface [2,3,4,5]. Thus, the substantial resistive losses accumulated in massive three-dimensional metamaterials are minimized using subwavelength-thick nanostructures. In addition, using the standard nanofabrication methods, such as photo and electron beam lithography, the metasurfaces can be easily fabricated [6,7].

Metasurfaces are a quickly growing research topic because of their unique capabilities in light manipulation by molding the wavefront, polarization, and nonlinear response of light in different ways. These types of optical metasurfaces of the first class can be used to manipulate the light at will, allowing the metasurface to transmit and/or reflect certain spectral components. These frequency-selective metasurfaces are very important because they can be used to design thin and efficient spectral filters that can be easily integrated with different imaging systems and replace the completely current interference and absorption color filters. Some examples can be found in the study of how light travels through nanoholes in metal films. It is also important to note that works on planar metamaterials with periodic or random resonant meta-atoms are significant. They can be adopted for both transmission and reflection, which can be very effective. There are a lot of exciting types of optical metasurfaces, but the ones that can control either the wavefront or the phase of light beams are the most interesting. These metasurfaces are made up of meta-atoms that are spread out in different places across the beam. They mostly use the geometric phase, also called the Pancharatnam–Berry (PB) phase principle, to obtain phase shifts in the range of 0–2π across different parts of the beam. These metasurfaces can be applied in areas, including metalens, holography, and beam shaping, leading to the replacement of numerous diffractive optical elements.

A wide range of geometric forms, dimensions, spatial orientations, combining mechanisms, and wavefront manipulation techniques go into rendering metasurfaces so versatile and adaptable in design. Due to their ability to manipulate the amplitude, phase, polarization as well as frequency of light in an irrational fashion, metasurfaces can be employed in a variety of applications, including holography [8,9,10,11], color printing [12,13,14], beam shaping and edge detection [15,16], polarization generation and detection, creation and exploitation of Terahertz waves, as well as optical encrypted communications anticounterfeiting technology [17,18].

In this review, we briefly discuss three fundamental mechanisms of wavefront modulation for metasurfaces. Next, we discuss the research progress of metasurfaces in the field of wavefront modulation and holographic displays, followed by novel research directions, such as metalens devices, cascaded metasystems, and vortex beam generation. The review is concluded with a summary of the potential challenges and future research directions of metasurfaces.

## 2. Principle of Arbitrary Wavefront Modulation for Metasurface

In the traditional optical and diffractive elements, the modulation of the incident beam was realized by collecting the phase delay, which significantly restricted the integration of optical components. Whereas in metasurfaces, the wavefront modulation is accomplished by controlling and modifying the amplitude and phase profile of the incident beam. These intriguing properties of optical metasurfaces provide some extra flexible design. Due to some strong interactions with the electromagnetic field, electric or magnetic resonances aid in wavefront control and allow nanoscale wavefront control. Modulating the phase distribution provides ultimate control over beam propagation, divergence, and information encoding. Several outstanding overview publications have highlighted the growth of wavefront engineering in recent years [19,20]. In this section, we introduce the three fundamental mechanisms for phase manipulation with particular polarization states, including plasmonic dispersion for linearly polarized light, the PB-phase for circularly polarized light, and Huygens’ principle for insensitive polarization [21].

### 2.1. Principle of Wavefront Engineering for Linearly Polarized Light

The classical laws of wave refraction and reflection can be changed in the presence of a metasurface [22,23,24]. By utilizing Fermat’s approach, Yu et al. reviewed generalized laws of reflection and refraction by introducing phase discontinuity at the boundaries shared by two mediums. Consider an incident plane wave with an incidence angle $\theta_{t}$. The presence of antennas provides an abrupt phase change. They expressed the relation between the incident and scattered waves as shown in Equation (1).

(1) $$\left\{\begin{array}{c} n_{t}\sin\left(\theta_{t}\right)-n_{i}\sin\left(\theta_{i}\right)=\frac{\lambda_{0}}{2\pi }\frac{d\left(\Phi \right)}{dx}\\ n_{i}\sin\left(\theta_{r}\right)-n_{i}\sin\left(\theta_{i}\right)=\frac{\lambda_{0}}{2\pi }\frac{d\left(\Phi \right)}{dx} \end{array}\right.$$

**Figure 1:**
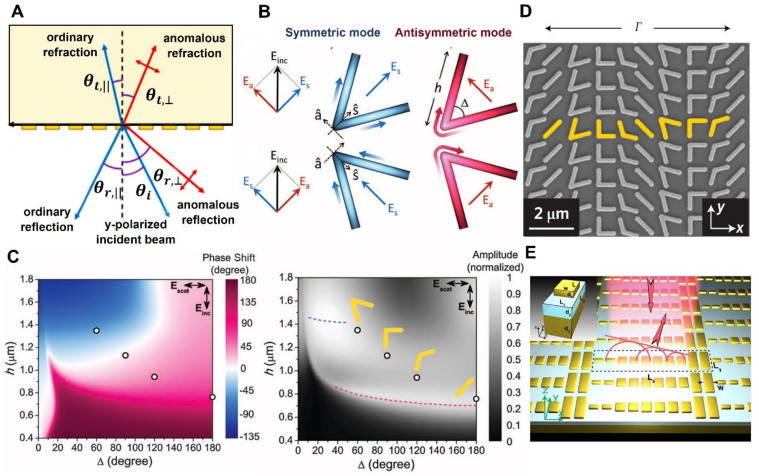
(**A**) Diagram of anomalous reflection and reflection beams from a metasurface with a phase gradient diagram for cross-polarized dispersed incident beams. (**B**) Both symmetrical and antisymmetric modes can be supported by V antennas, incident field components â and ŝ axes. The antenna’s symmetry axis and the angle of incidence are both 45 degrees. Blue antenna color for symmetric and red colors to demonstrate the antisymmetric mode showing the schematic current distribution, with stronger colors indicating high currents. Arrows with a color gradient show the direction of the current flow [23]. (**C**) Computed amplitude and phase shift of C.P. distributed light considering V-shaped nanoantennas with various varied lengths and opening angles. The four circles shown in (**C**) illustrate the length (h) and opening angle (Δ) values utilized in tests. The rod shape allows for analytical calculations of the phase and amplitude of scattered light without the need for the expensive numerical simulations required [23]. (**D**) Scanning electron microscopy images of the plasmonic interface unit cell (yellow), made up of eight gold V-antenna arrays fabricated on a silicon wafer [23]. (**E**) Near-infrared gap-plasmon metasurface with a background plane causes anomalous refraction phenomena. A Magnesium Fluoride (MgF_2_) spacer separates a gold nanorod from a gold film, as shown in the left inset, the fundamental building component [34]. (**B**–**D**) Reprinted/adapted with permission from Ref. [23]. Copyright 2011, American Association for the Advancement of Science (AAAS). (**E**) Reprinted/adapted with permission from Ref. [34]. Copyright 2012, American Chemical Society (ACS).

The abrupt phase discontinuity presented at the interface is shared by two mediums with refractive indices $n_{t}$ and $n_{i}$. As illustrated in Figure 1A, the angles of transmitted and reflected waves are denoted by $\theta_{t}$ and $\theta_{r}$, respectively. Any photonic device’s surface can be used to change the propagation direction of refracted and reflected rays, and this can be done by using an ultra-thin layer. Equation (1) suggests that both the refracted waves and reflected waves may occur in any direction, as long as an appropriate continuous gradient of phase discontinuity along the interface $\frac{d\left(\Phi \right)}{dx}$ is present.

Using the phase discontinuity, one can achieve the control of anomalous reflection and refraction and steer the light by the generalized Snell’s law. A combination of two or more resonant nanorods with varying rotated lengths can be used to manipulate the phase over the whole 2π phase range. It is preferable for rotating nanorods to employ circularly polarized input and output waves since the wave’s amplitude is unaffected by metasurface transmission. The first proposed nanostructure was V-shaped antennas made of two nanorods of equal length connected at a specific angle. According to the current distributions, V-shaped antennas support “symmetric” and “antisymmetric” modes, which are stimulated by electric-field components along with the $\hat{s}$ and $\hat{a}$ axes, respectively, as illustrated in Figure 1B. The plasmonic nanorods experience a rapid phase shift with different amplitudes by varying their length and opening angle, as demonstrated in Figure 1C. The physics of the V-shaped-based antenna and its systematic model were explored in great depth by Capasso’s group. The crossed polarized scattered light can possess a wide range of phases and amplitudes achieved by altering the antenna geometry, orientation, and antenna polarization as first proven by Yu et al. and subsequently demonstrated by Ni et al. in mid-infrared and near-infrared, respectively [25,26]. The antennas in the array are shaped and oriented so that they exhibit a variety of resonant properties. The array’s design and orientation are set such that the component of the scattered field polarizer in the 90-degree direction has an incremental phase of π/4 across consecutive V-antennas for a typically incident field polarized in a direction orientated at an angle α from the x-plane, as shown in Figure 1D. Referring to the idea of a gradient metasurface, various alternative structures, such as C-shaped antennas [27], nanorods [28], and nano bricks [29], are developed subsequently and provide complete phase control capacity. Moreover, by altering the dimensions of the unit cell, comparable E.M. responses were produced in several frequency ranges, including visible [30], near-infrared, and mid-infrared frequencies [1,23]. These suggested designs were accomplished via cross-polarized components connected to the scattering field. The greatest coupling efficiency between the two polarizations was severely restricted inside a single metasurface, and therefore, only a tiny quantity of incoming electromagnetic fields may engage with the metasurface while a considerable amount of energy radiated outwards in a usual fashion [31]. The efficiency can be boosted by gradient metasurfaces functioning in reflection with a background plane, as illustrated in Figure 1E [32,33]. This type of metasurface is composed of metal-insulator-metal (MIM) structures, in which nanoantenna arrays were isolated from a metallic ground film by a thin insulator layer. High coupling between the top antenna layer and the metallic ground plane induced magnetic resonances enabling a phase delay of up to 2π to be accomplished [34].

### 2.2. Modulation of the Wavefront of Circularly Polarized Light through Pancharatnam-Berry Phase

**Figure 2:**
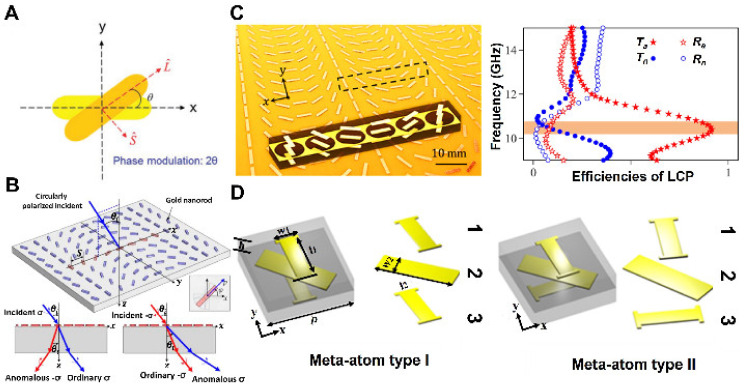
(**A**) Schematic illustration of PB phase metasurface using nanorod structure; a phase response is exclusively dictated via the orientation of nanorod θ against the *x*-axis [3]. (**B**) Schematic pictures of ordinary and anomalous refraction based on PB metasurface once illumined with polarized LCP or RCP light [39]. (**C**) Illustration of designed PB metasurface demonstrating electric and magnetic responses (**right**) along with their transmissive conversion efficiency with respect to frequency (**left**) [43]. (**D**) The suggested meta-atoms, referred to as meta-atom I and meta-atom II, are made up of tri-metallic layers surrounded by dual F4B substrates [44]. (**A**) Reprinted/adapted with permission from Ref. [3]. Copyright 2017, Wiley-VCH. (**B**) Reprinted/adapted with permission from Ref. [39]. Copyright 2012, American Chemical Society (ACS). (**C**) Reprinted/adapted with permission from Ref. [43]. Copyright 2017, American Physical Society (APS). (**D**) Reprinted/adapted with permission from Ref. [44]. Copyright 2017, Wiley-VCH.

Pancharatnam–Berry (PB) phase is another technique that attracted intense attention because of its strong capabilities in modulating the CP wave. In the PB phase, the variations in phase or amplitude of the metasurface are created by varying the geometry of antennas, and the 2π phase control can be accomplished by adjusting the orientation angle of identical geometry antennas. Assume an anisotropic nanostructure with normal incidence when the polarization of incident light along the nanostructure’s two principal axes, where $t_{0}$ and $t_{e}$ represent its complex scattering coefficients, respectively. When the nano resonator is rotated at an angle from the x-axis, as illustrated in Figure 2A, the scattered coefficients for the rotating system may be determined using the Jones matrix operation; [35,36].

(2) $$\begin{array}{c} \hat{t}\left(\theta \right)=R\left(-\theta \right)\left(\begin{array}{cc} t_{0} & 0\\ 0 & t_{e} \end{array}\right)R\left(\theta \right)\\ =\left[\begin{array}{cc} \cos\theta & -\sin\theta \\ \sin\theta & \cos\theta \end{array}\right]\left[\begin{array}{cc} t_{0} & 0\\ 0 & t_{e} \end{array}\right]\left[\begin{array}{cc} \cos\theta & \sin\theta \\ -\sin\theta & \cos\theta \end{array}\right]\\ =\left[\begin{array}{cc} t_{0}\cos^{2}\theta +t_{e}\sin^{2}\theta & (t_{0}-t_{e})\cos\theta \sin\theta \\ (t_{0}-t_{e})\cos\theta \sin\theta & t_{0}\sin^{2}\theta +t_{e}\cos^{2}\theta \end{array}\right] \end{array}$$

where $R\left(\theta \right)$ is the rotation matrix. The scattered CP can be calculated for the incident circularly polarized (CP) by matrix multiplying Equation (2) by the Jones’ vectors of left-handed circularly polarized (LCP) or right-handed circularly polarized (RCP) light ${\hat{e}}_{L/R}=({\hat{e}}_{x}\pm i{\hat{e}}_{y}/\surd 2$) and the scattered CP $\left(Es\right)$ can be expressed as [37,38]:

(3) $$Es=\hat{t}\left(\theta \right).{\hat{e}}_{L/R}=\frac{\left(t_{0}-t_{e}\right)}{2}{\hat{e}}_{L/R}+\frac{\left(t_{0}-t_{e}\right)}{2}\exp\left(\pm i2\theta \right){\hat{e}}_{R/L}$$

It can be realized from the above Equation that scattered light is comprised of two circular polarization states. The first component in Equation (3) denotes CP scattered waves with the same helicity as the incident wave without any phase delay, while the second denotes CP scattered waves with the opposite helicity carrying a different PB phase (*±i*2*θ*). Moreover, Huang et al. came up with a novel solution to the problem of metasurface dispersion. They illuminated that antenna elements made of nanorods with circularly polarized light were used [39]. Orientation of the nanorod determines the phase shift in the opposing helicity field component when such an antenna disperses the radiation. The phase delay of the incident LCP and RCP is expressed by the “+” and “−” signs, as illustrated in Figure 2B [39]. This technique utilizes aligned copies of a single antenna design based on the PB phase principle for phase modulation and can be applied for broadband applications without imposing an additional fabrication burden. The linear relation of phase delay to the orientation angle of the scattering elements provides the phase control from 0 to 2π by rotating the nano-resonator from 0 to π [40], such as C-type structure resonators, nanorods, and nano-slits [29,41,42]. While PB phase metasurfaces can control CP beams, early published studies found that the conversion efficiency was very low using PB phase metasurfaces. The highest conversion was restricted by 25% using a plasmonic single-layer ultrathin PB phase [31]. Moreover, metal-insulator-metal (MIM) structures functioning in a reflected way were presented in order to boost working efficiency and were promising. It is also possible to produce an ultra-high efficiency design based on an ultra-thin PB metasurface that incorporates the electric as well as magnetic resonators, subsequent in both constructive and destructive interference on the transmission and reflection side, as illustrated in Figure 2C [43].

A novel design for CP bifunctional metasurfaces was presented by Tong et al. by utilizing the PB meta-atoms with helicity-sensitive transmissions in addition to the reflections. Two types of meta-atoms were proposed, as shown in Figure 2D, that can be used as meta-lens and CP beam splitters, bringing up new ways to expand further ultra-high performance multifunctional metasurfaces in other frequency domains [44].

### 2.3. Huygens’ Metasurfaces

**Figure 3:**
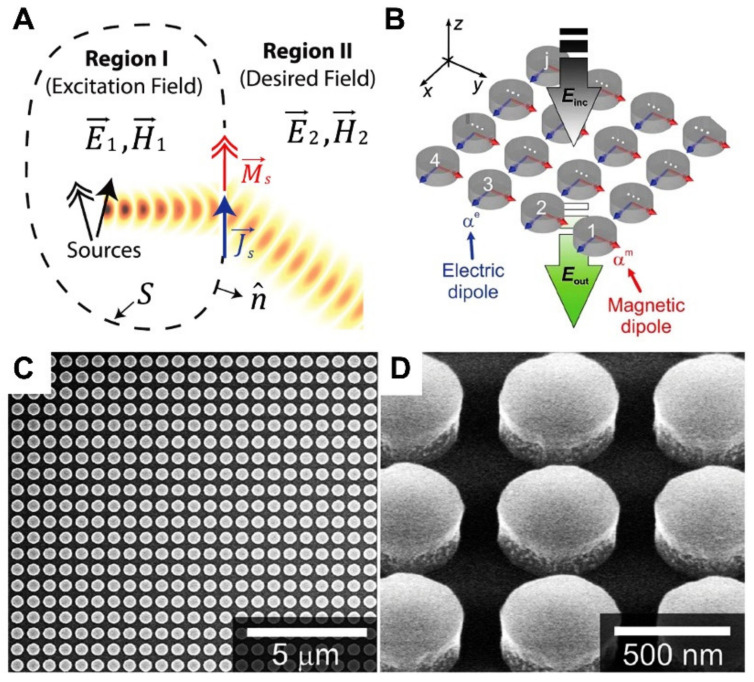
(**A**) With the appropriate arrangement of surface electric and magnetic currents provided by the equivalence principle, desired field distributions satisfying the Helmholtz equation are independently maintained in excitation and desired regions [51]. (**B**) Schematic of infinite arrays of nanodisc representing Huygens’ metasurface with electric and magnetic dipole polarizabilities αe and αm for incoming x-polarized light [46]. (**C**) Illustration of a top view and (**D**) side view of nanodisc meta-atom made of silicon [46]. (**A**) Reprinted/adapted with permission from Ref. [51]. Copyright 2013, American Physical Society (APS). (**B**–**D**) Reprinted/adapted with permission from Ref. [46]. Copyright 2015, Wiley-VCH.

At optical frequencies, plasmonic metasurfaces suffer high dissipative losses, along with numerous loss channels, including diffraction, ordinary reflection/refraction, and polarization conversion loss, all of which occur during the phase-modulation process. Therefore, researchers are exploring the dielectric analogs of metasurfaces. Huygens’ metasurfaces, for example, were employed to attain perfect transmission by forming high-refractive-index dielectric nanoparticles or nanodiscs. The suggested design potentially generates spectrally coinciding electromagnetic resonating responses with comparable strength [45,46,47]. High-refractive-index dielectric nanoparticles with minimal losses can link incident waves to induce circular displacement currents inside nanostructures, resulting in a significant magnetic dipole resonance shown in Figure 3A.

In contrast to their metallic equivalents, substantial metallic losses at visible frequencies cause fields within nanoparticles to vanish, leading to a weak magnetic response [7,48,49,50]. According to the surface equivalent concept, Huygens’ metasurface considers the impedance match on an interface that is polarization-independent and simultaneously stimulates the dipole moments of both electric and magnetic fields. Observation of Figure 3A shows that the surface equivalence principle dictates that the proper distribution of fields and boundary conditions are only possible at the interface when both electric and magnetic surface currents are present [51]. Consequently, field discontinuities need compensating using the following relation:

(4) $$\begin{array}{c} \vec{J_{s}}=\hat{n}\times \left(\vec{H_{2}}-\vec{H_{1}}\right)\\ \vec{M_{s}}=-\hat{n}\times \left(\vec{E_{2}}-\vec{E_{1}}\right) \end{array}$$

where $\vec{J_{s}}$ and $\vec{M_{s}}$ represent surface electric and magnetic currents, respectively. In most cases, the carefully designed subwavelength metallic pattern was manufactured on a dielectric substrate in order to obtain the necessary surface impedance of the individual unit. It was claimed that a universal approach could be used to transform the transmission/reflection phase distributions that are needed for the appropriate surface profiles for Huygens’ metasurface [52]. High contrast transmit/reflect arrays were used, and a large high index layer was utilized to develop this technology to minimize the amount of transmission loss [53,54]. The high ohmic loss of the metallic metasurface, particularly in the optical frequency region, not only constraints the practical application but also degrades the performance. Therefore, the dielectric metasurface with low loss has gained research interest [30,55,56,57]. Kivshar’s group physically demonstrated a complete phase coverage from 0 to 2π with transmission efficiency above 50% in the near-infrared region using silicon nanodisc. The schematic and electronic micrograph images of silicone nanodiscs are illustrated in Figure 3B,C [46,58]. Moreover, a beam deflector created on all-dielectric Huygens’ metasurfaces was similarly developed, with an efficiency of more than 45% observed in the visible spectrum [57].

In contrast to the plasmonic resonance in metallic, the electric and magnetic resonances that are contributed to by the dielectric metasurface can be categorized as Mie resonances [50,59,60,61]. These resonances can produce amplitude and phase modulation [53,62].

## 3. Potential Application: Wavefront Shaping Engineering

Since the emergence of the metasurface, it has shown exceptional wavefront engineering capabilities, ushering in a wide range of promising applications in various fields. After categorizing the theory and methods of the metasurface to regulate the phase and amplitude under various polarization states, a full examination of their applications in the realm of photonics devices is being explored in this section.

### 3.1. Planar Meta-Lenses

**Figure 4:**
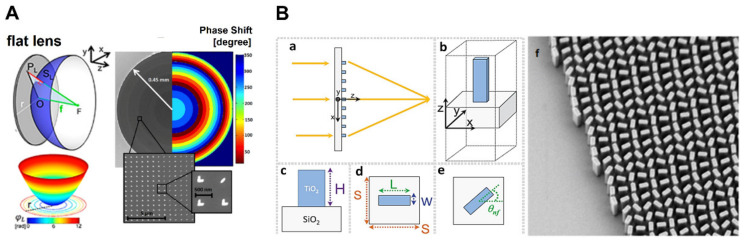
(**A**). Schematic illustrating the sample of a planar lens, the phase shift at a point P_L_ associated with a surface of the lens is proportionate to the P_L_S_L_ distance (**left**). SEM picture of planer lens along with phase shift distribution estimated from Equation 6 and discretized into eight equal antennas (**right**) [66]. (**B**) (**a**) Diagram of the TiO_2_ nanofin-based dielectric metalens and its constituent. (**b**) TiO_2_ nanofins on a substrate material make up the metalens. (**c**) Side view, (**d**) top view of the nanofin’s dimensions z span (H), y-span (W), and x-span (L). (**e**) Rendering the geometric PB phase, the requisite phase is imparted by rotating the nanofin via angle $\theta_{nf}$. (**f**) SEM images of the metalens were taken at a scale bar of 300 nm [70]. (**A**) Reprinted/adapted with permission from Ref. [66]. Copyright 2012, American Chemical Society (ACS). (**B**) Reprinted/adapted with permission from Ref. [70]. Copyright 2016, American Association for the Advancement of Science (AAAS).

Metasurfaces can deliver abrupt phase changes to incoming electromagnetic fields within sub-wavelength scales. The light beam can be twisted and focused, and unique optical beams such as optical vortex and Bessel beams can be generated using the metasurface’s controlled phase profile [63,64,65]. The fundamental technique utilized throughout the years to regulate the form of the incoming wave is phase profile engineering. A full description of the production of the phase profile for meta-lens is provided in the following section. To build flat lenses based on a metasurface, a phase profile should satisfy the given Equation (5) to convert the incident planar into a spherical one that converges at a distance f from the lenses [66].

(5) $$\Phi \left(x,y\right)=2\pi /\lambda \sqrt{x^{2}+y^{2}+f^{2}}-f$$

where λ denotes the wavelength in free space. Based on the principle of phase discontinues, a nanoantenna meta-lens was developed by Capasso et al. at a telecom wavelength utilizing V-shaped nanoantennas, as illustrated in Figure 4A, with a focusing efficacy of just 1% [66]. Flat lenses with complementary V-shaped spaces were also presented in the optical spectrum, although the efficiency was not promising [25]. These metalenses have reported low efficiency because of the restricted light coupling capability to plasmonic antennas comprising the single layer, and the concentration was on the CP scattering element [66]. In addition, Capasso and his fellow researchers developed the concept of the achromatic meta-lens, which has the ability to correct the dispersive accumulated phase that happens as a result of light propagation and has a wavelength-dependent phase contribution. They constructed an achromatic metalens that worked at telecom wavelengths by using an aperiodic design and consisting of connected rectangular dielectric resonators [52]. As a result of the design, incident near-infrared light with wavelengths of 1300, 1550, and 1800 nm can all have identical focal lengths [67].

Recently, planar lenses based on the PB-phase approach have also been demonstrated using nanorods and U-shaped apertures [19,68,69]. The mandatory phase distribution is obtainable when CP incoming light is transformed to the opposite helicity. It has been shown that metalenses with an 86% efficiency can be made by employing titanium dioxide (TiO_2_) nanofins that have the rotational structure of the “PB” type shown in Figure 4B [70]. It was then utilized to design two interlaced arrays of TiO_2_ building blocks with opposing helicities to demonstrate an imaging system that is chirality-distinguishable [71]. The inherent dispersive behavior of the resonators in many metasurface-designed lenses causes significant chromatic aberrations, decreasing an imaging system’s performance, much as it does in conventional lenses owing to the dispersion of the phase gathered by light through propagation [72,73]. A comparable combination method was used by Yu et al., and they were able to achieve an insensitive polarization metalens with a focusing efficiency of above 20% within a bandwidth of 1200–1650 nm [74]. Additionally, an achromatic metalens with an efficiency of 12% was accomplished experimentally by using a low-quality factor resonance generated phase and geometric phase that recompense for one another [75].

With an average efficiency of around 40%, a GaN-based meta-atom-based integrated resonator was proposed to reduce chromatic aberration at wavelengths (400–660 nm) that are not visible to the naked eye [76]. Tunable dielectric meta-lenses have also recently been suggested in order to increase the number of meta-devices that can be used with more flexibility [55,77]. These reconfigurable metasurfaces are discussed in more detail later in this study.

### 3.2. Metasurface Holography

**Figure 5:**
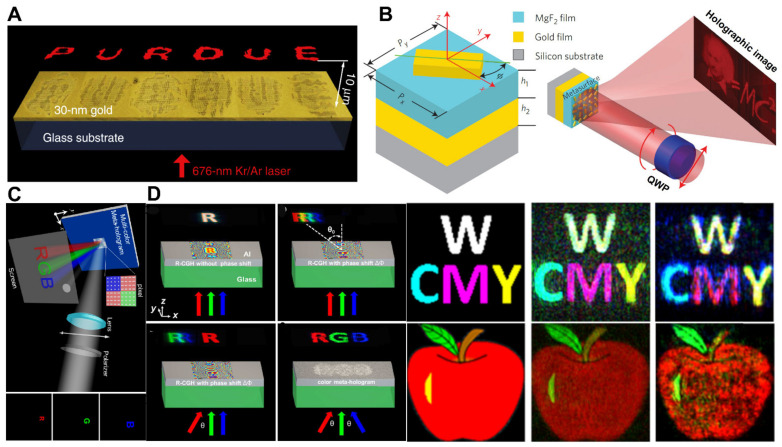
(**A**) Experimental metasurface holograms create holographic pictures. The word ‘PURDUE’ is shown in the picture. Kr/Ar visible laser illumines the model from the glass substrate side at 676 nm. A Babinet-inverted, complementary plasmonic antenna schematic view (**right**) [79]. (**B**) The unit cell structure of a nanorod-based hologram with a cell structure is shown. Different phase delays can be achieved by moving the nanorod in the *x*–*y* axis at an angle of ϕ (**right**). Demonstration of CGH based on nanorod structure. In this method, a metasurface is illuminated by the incident CP beam, passing through a quarter-wave plate (QWP). In a distant field, the holographic picture is formed by the reflected beam (**right**) [80]. (**C**) A linearly polarized image of the multicolor meta-hologram (MCMH) is illustrated. In the MCMH structure, aluminum nanorods form pictures R, G, and B at particular wavelengths. Moreover, a SiO_2_ spacer sprayed on an aluminum mirror is used to shape the pixels. Rebuilt pictures of letters R, G, and B and placements relative to the 0th order point in the top right corner of the display screen, to make the recreated pictures fit into appropriate spatial order and seem equal in size [82]. (**D**) Metasurface holograms in which three main color components can be decoded using an off-axis illumination approach and their secondary superimposed colors can be generated [85]. (**A**) Reprinted/adapted with permission from Ref. [79]. Copyright 2013, Nature Publishing Group (NPG). (**B**) Reprinted/adapted with permission from Ref. [80]. Copyright 2012, Nature Publishing Group (NPG). (**C**) Reprinted/adapted with permission from Ref. [82]. Copyright 2015, American Chemical Society (ACS). (**D**) Reprinted/adapted with permission from Ref. [85]. Copyright 2016, American Chemical Society (ACS).

The concept of holography was originally introduced by Denis Gabor in 1948 and is one of the most intriguing imaging techniques in wave phenomena, allowing the recording and reconstruction of the whole three-dimensional light field of a specific target object. Metasurfaces are capable of creating holograms by tailoring and reshaping the amplitude and phase of EM waves [78]. Researchers such as Shalaev et al. developed a complementary V-shaped groove antenna manufactured in a gold layer on a glass substrate and exhibited a holographic image “PURDUE” lit via a visible laser in 2013, as illustrated in Figure 5A [79]. Based on PB configuration with the assistance of metallic nanorods, a 3D holographic image with a large field of view (FOV) of 40° was experimental realized [28]. Unlike one-layered plasmonic metasurfaces, which are generally inefficient, the metal-insulator-metal (MIM) reflection configuration was used to reconstruct a holographic image of Einstein’s portrait at a wavelength of 825 nm with promising efficiency (80%), as illustrated in Figure 5B [80]. In order to improve the transmissive metasurface holograms, a dielectric Huygens’s metasurface has experimentally proved a promising diffraction efficiency of over 99% using a silicon nanopillar designed at a working wavelength of 1600 nm [81]. However, as mentioned earlier, all of these metasurfaces were for monochromatic holograms, where the meta-atoms were made for a particular wavelength. By putting multiple resonators with different wavelengths of resonances into each meta-unit cell, metasurface designs can control beams of more than one wavelength at the same time. Tsai et al. demonstrate this methodology and grasped binary phase hologram function for colors, i.e., red, green, and blue, as given in Figure 5C [82].

Another example is the design of super-unit cells by Choudhury et al. that include tri-silver nano-slits of varying dimensions. Thus, a three-color hologram can be created [83] and transformed into full-color holograms by modulating the phase and intensity of primary color lasers. This process is known as “color mixing”. Three color components can be decoded and produce their secondary superimposed colors by the color mixing process [84,85]. Recently Wan et al. and Li et al. utilized an off-axis lighting approach, which uses red, green, and blue laser beams to illuminate metasurfaces with different tilted incidence positions, as shown in Figure 5D (left). The simulated and experimentally reconstruction of 2D images of holograms with primary and their secondary colors are demonstrated in Figure 5D (right) [85]. Holographic images were created utilizing dielectric metasurfaces, such as titanium dioxide (TiO_2_) and graphene oxide nanodiscs, to produce full-color images [86,87]. Metasurfaces that can modify phase, amplitude, and polarization simultaneously and independently have recently been suggested. By integrating the frequency-independent PB phase with the detour phase, Li’s group was able to build a full-color meta-hologram [88]. It is still worth exploring several interesting aspects based on metasurface holography techniques that are blooming, including holographic detection and measurement, augmented reality (AR), virtual reality (VR), and imaging.

### 3.3. Cascaded Metasystem

In this section, we discuss recent developments in metasurfaces as building blocks for metasystems. Our definition of a “metasystem” includes unique configurations of numerous metasurface layers intended to give capabilities not feasible with a single layer of the metasurface. High-performance and economical optical systems can be created by integrating planar wavefront metasurfaces vertically using monolithic techniques. Since no post-fabrication alignments are required, the optical systems are immediately connected with optical sensors and optoelectronic components, allowing for low-power and compact micro-optical systems. In this section, examples of contemporary meta-system applications are discussed.

#### 3.3.1. Reconfigurable and Tunable Metasurface

The characteristics of all of the aforementioned metasurface devices are typically static. It is extremely desired to be able to tweak, alter, or reconfigure the operation of these optical components. Therefore, the researchers have conducted a lot of work to make the metasurfaces tunable and reconfigurable with respect to changes in operational conditions. Several practicable approaches were anticipated and explored, such as mechanical deformations and re-configurations [77,89,90,91,92], electrical and magnetic tuning [93,94,95,96,97,98,99,100,101,102], thermal tuning [97,103,104], and material re-configuration using phase-change materials [105,106,107]. Mechanically adjustable metasurfaces on flexible substrates are one of the potential tuning platforms that have allowed varifocal lenses [77,89,107], color tuning [108], and frequency response tuning [109].

Tunable metalenses and meta-holograms are other applications of tunable metasurfaces. Numerous studies have been carried out on tunable metasurfaces by engineering the optical responses via various incident lights, varying the configuration of met-atom, or changing the relative distance between adjacent metasurfaces. In this overview, we go over the basics of metalenses and meta-holograms. The majority of tunable optical responses are achieved by changing the light sources or applying a voltage; therefore, the tuning techniques we categorize are (1) light source control, (2) electrical tuning, and (3) non-electrical tuning. For example, phase change materials (PCM), mechanical deformation, and changes in the relative location of cascaded metasurfaces are examples of non-electrical tuning.

(1) Light Source Control Tuning Approach

**Figure 6:**
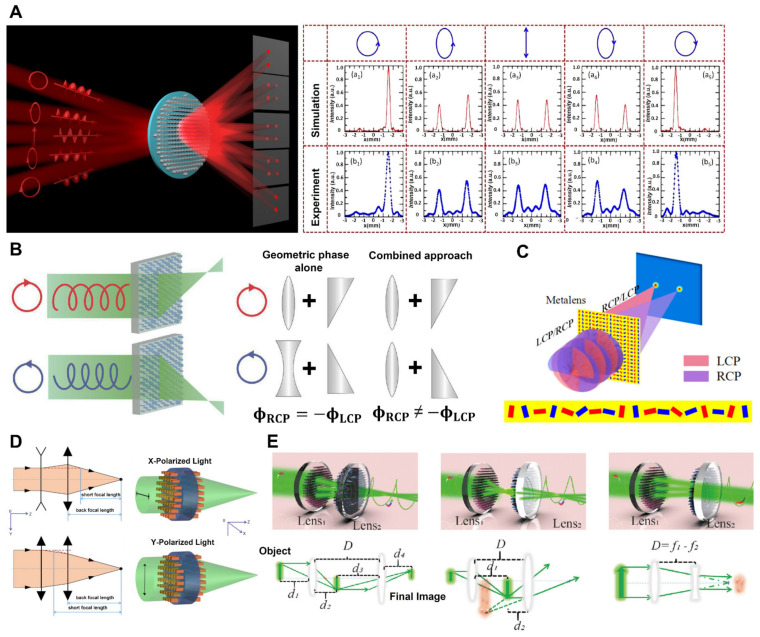
Tunable metalenses based on light source: (**A**) Demonstration of spin-decoupled metalenses in a schematic form with various intensity tunable multiple focal points (**left**). Simulated and experimental electric field intensity profile at line y = −1.5 mm for different incoming light polarization states (LCP, LECP, LP, RECP, RCP from left to right) (**right**) [110]. (**B**) The chiral meta-lenses are working. RCP light can illuminate a meta-lens, resulting in an RCP—LCP image focused on two different places (**left**). Comparison of fundamental design differences between prior chiral lenses (**right**). The phase function of an RCP geometric phase lens is equal to and opposite to that of the LCP. A refractive lens and a wedge are used in this example to focus RCP to the right and defocus LCP to the left. LCP and RCP may be independently focused using the propagation and geometric phase combination approach [111]. (**C**) Schematic of focusing in various places utilizing the developed 93 spin-selected metalenses for circularly polarized light [112]. (**D**) Schematic of metalenses utilizing the PB and propagation phases at various locations [113]. (**E**) Schematic design of multifunctional polarization-dependent metal lens doublets [114]. (**A**) Reprinted/adapted with permission from Ref. [110]. Copyright 2021, Optica Publishing Group (OPG). (**B**) Reprinted/adapted with permission from Ref. [111]. Copyright 2018, Springer Nature. (**C**) Reprinted/adapted with permission from Ref. [112]. Copyright 2018, IOP Publishing. (**D**) Reprinted/adapted with permission from Ref. [113]. Copyright 2019, Optica Publishing Group (OPG). (**E**) Reprinted/adapted with permission from Ref. [114]. Copyright 2020, Wiley-VCH.

Tunable metalenses can be used to adjust the feature of a light source, such as polarization. The intensity of the focal point in these metalenses can be controlled by changing the circular polarization state of the incident beam. Moreover, the generation of multiple focal points can be grasped by the ellipticity of the incident beam. Spin-decoupled metalenses were created by combining the characteristics of many convex and concave lenses into a single metasurface utilizing the PB phase. One phase profile focuses on incoming LCP light, while the other profile focuses on incoming RCP light [110]. As a result, the focus point shifts as the polarization of the incoming light changes; Figure 6A (left). Furthermore, by adjusting the ellipticity of the input light, the intensity of various focus sites may be controlled, as illustrated in Figure 6A (right). It is easier to construct a spin decoupled metalens utilizing just the geometric phase rather than both the propagation and geometric phases since there is no need to scan many parameters. However, the recommended metalens provides 50% efficiency, which is not promising. The light at two different focal points can be focused by the metalens depending on the spin state of the input light. The PB phase implements the required phase profile in a silicon nano brick unit structure. When the spin state of the incoming light changes, two silicon nano bricks (red and blue on the metalens) function as a curved lens or a bowl-shaped lens, as shown in Figure 6B [111]. These spin-selected metalenses may be used for spin-controlled photonics and detection techniques. Moreover, an example of a step-zoom metalens is demonstrated in Figure 6C [112]. The focal distance varied with respect to the polarization of the incoming light. A metalens with two focal distances utilizes metasurfaces on both sides. When the y-polarized beam is incident, both metasurfaces function as convex lenses, while in the case of x-polarized light, one metasurface works as concave, and the second functions as convex, as demonstrated in Figure 6D [113]. This type of metalens design approach is compact, simple, and flexible and has promising potential for application in the field of bioengineering, optical communication, and wearable electronics technology. Moreover, it has been claimed that a metalens doublet can accomplish different functions depending on the polarization of the incoming light and the distance between the two lenses. Changing the incoming beam’s CP state and the distance among the lenses can perform as a three-function doublet, as illustrated in Figure 6E [114]. Since no separate optical components are employed for multifunctional metalens doublet, it simplifies and reduces the size of the imaging setup. Consequently, it has a lot of potential for use in compact imaging devices.

(2) Electrical Tuning Approach

Tunable metalenses can also realize by applying an external electrical bias voltage across active materials, including graphene [115,116] and liquid crystals (LCs) [117,118]. For example, in transmission-type terahertz metalenses, the chromatic aberration tunability can be realized by combining a dielectric metasurface with LCs. When an external voltage source is applied across the LCs, their geometric phase modulation disappears and leaves just the resonant phase in the metalens, which changes the function of the device from achromatic to dispersive focusing. This type of tuning can provide dual functions from one metalens. Moreover, a varifocal metalens is another example of combined metalens that tunes the numerical aperture by keeping the twisted nematic LCs below the metalens’ substrate. Based on the applied voltage bias, the device converts the polarization state of incoming light and provides different focal points [119]. Moreover, variable focal length lenses (varifocal) based on flexible plasmonic metasurfaces are formed by a geometric phase that only works for one CP. Furthermore, similar varifocal lenses can be electrically actuated using electrically elastomers [90]. These types of metalenses have great potential application in the field of biomedical and optics because of their high-quality image and high-speed switching response.

Graphene, a 2D material, has been extensively used in the realization of an electrically tunable metasurface in the THz and mid-IR regime, thanks to its wide range tunability for electrical conductivity. Graphene tuneability is achieved with focal distance depending on the applied external biasing voltage to enable the fermi level and charge carrier to change. As a result, the absorption and transmittance of graphene are varied. Consequently, the phase tuning capabilities of these graphene metasurfaces are relatively restricted (usually considerably less than π), severely limiting their potential for dynamic wavefront modulation.

(3) Non-electrical Tunning Approach

A rotationally-moved tunable deflector is an example of a mechanically tuned metasurface, controlled by rotating the two metasurface layers in opposite directions. It can be employed as a laser scanning device in fields such as optical communication, measurement, bio-imaging, and display [120]. Additionally, mechanical motions of numerous rigid nanostructures were also examined for tuning the focal length via utilizing the Alvarez optical lenses. In terms of thermal tuning, the metasurface changes its optical properties when changes occur in the external temperature. Recently, different PCMs have been employed, such as vanadium dioxide (VO_2_) and graphene, opening up numerous applications in optical sensors and thermal tunable devices [121].

Despite the significant advances made in the area of tunable and reconfigurable metasurfaces, many major challenges are still unresolved. For instance, to flexibly regulate the EM waves dynamically, one must modify both the phase and amplitude of the local EM field. However, several current known techniques to manipulate the phases and amplitudes of EM waves concurrently and inaccessibly severely limit their usefulness in developing functional tunable metalens. Thus, novel techniques that can achieve wide-range yet decoupled modulations on the EM phase and amplitude, especially at high frequencies, are highly demanded. Developing new fabrication technologies and compatibility issues with modern semiconductor technology are challenges that provide new research directions for researchers.

#### 3.3.2. Cascaded Metasurface-Based Retroreflector, Spectrometer

**Figure 7:**
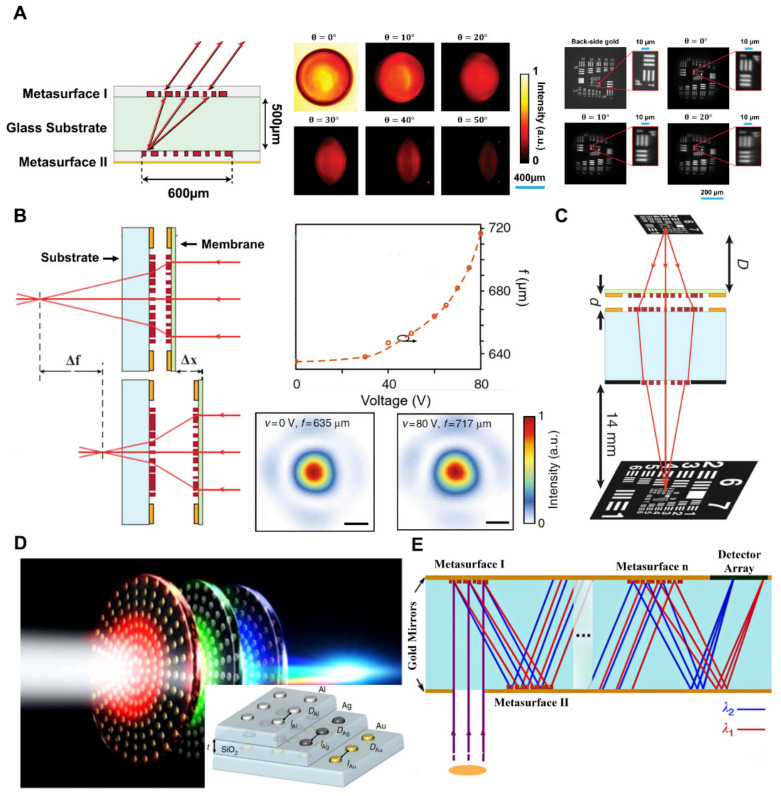
(**A**) Schematic illustration planar retroreflector consists of bi-metasurfaces deposited on opposite sides of the glass substrate (**left**) [124]. The device’s measured reflectance as a function of incoming angle brightness intensity (middle). Images of the entity in the back-side gold mirror and the retroreflector were calculated based on the angle of rotation retroreflector (**right**). (**B**) Tunable dielectric lens comprises two metalenses cascaded at a variable distance. One lens is fixed on a substrate, and the second one is moving on a membrane (**left**) [55]. The calculated front focal length against input DC voltage (**right top**) and at various actuation voltages, intensity distributions in the focal plane of the doublet lens (**right bottom**). (**C**) Displays that adding an aperture in a single tiny composite metadevice, metalens in front of a focusing metalens, can accurately correct the aberrations and accomplish the functions of a Chevalier landscape lens with a resolution near the diffraction limit [55]. (**D**) The three-layer lens depicted in this illustration. Each layer’s assigned spectrum portion is targeted to a length of 1 mm along the optical plane when lit with white light. The layered structure schematic is shown in the inset (images captured using a scanning electron microscope [125]. (**E**) An ultrafine spectrometer consists of several folded metasurfaces. Even more intriguing, rather than a straight vertical stack, metasurfaces might be folded serially inside the optical path. In the case of numerous metasurfaces, this method may further decrease the device footprint, resulting in spectrometers with ultrafine resolution and extremely thin thicknesses [126]. (**A**) Reprinted/adapted with permission from Ref. [124]. Copyright 2017, Springer Nature. (**B**,**C**) Reprinted/adapted with permission from Ref. [55]. Copyright 2017, Springer Nature. (**D**) Reprinted/adapted with permission from Ref. [125]. Copyright 2017, Springer Nature. (**E**) Reprinted/adapted with permission from Ref. [126]. 2018, Springer Nature.

A miniature planer metasurface retroreflector was implemented by assembling dual flat metasurfaces vertically, one executing spatial Fourier transform and its inverse, and the other conveying a spatially variable velocity to the incoming light’s Fourier transform. Under normal incidence, 78% efficiency was measured with 60° FOV. It should be noted that making a retroreflector that can operate across an extensive range of angles using a one-layer metasurface is impossible [53,122,123]. A schematic illustration planar retroreflector is shown in Figure 7A. The SEM images of the manufactured device are demonstrated in Figure 7A (middle), and the experimental focal distances and in-focus intensity profiles are illustrated in Figure 7A (right) [124]. Tunable doublet metalenses have recently been fabricated using the MEMS and metasurfaces shown in Figure 7B (left). Changing the distance between two metalenses by a short distance (~1 nm) is enough to tune the entire focal length by a substantially more significant amount (~30 nm). In order to show an ultra-compact microscope (~1 mm), a comparable adjustable metasurface doublet is paired with another metasurface printed on the opposite side of the glass substrate, as shown in Figure 7C. With a working distance from 5 to 13 µm, the microscope has a broad corrected field of vision (500 um, or about 40°) [55]. Moreover, the use of nanostructure metasurface in multi-wavelength or broadband applications is limited due to strong chromatic aberrations. To reduce chromatic aberrations in specific diffractive components, numerous insensitive metasurfaces are stacked vertically, each comprised of a variety of materials and optimized for one specific spectral frequency range. Three-layer lens achromatic metalens are utilized in the visible range, as shown in Figure 7D. Further on, a self-aligned embedded component is also demonstrated for spontaneous emission depletion imaging with the optical lens to focus on anomalous dispersive. These findings open a path for multifunctional ultra-thin super achromatic optical components of nanostructured metasurfaces [125]. Recently a technique of a folded tiny spectrometer is presented as shown in Figure 7E. The reflective metasurfaces on both sides of a visible substrate are used to achieve all dispersive and focusing optics. Reflectors across both sides of the substrate confine as well as guide the light propagation within the substrate. Moreover, the sensor can be placed at device’s yield aperture directly [126]. Furthermore, a nanofins-based metalens doublet is demonstrated by the researcher, capable of performing diffraction limiting focusing with a numerical aperture (0.44), a focal length (342.5 m), and 50° and providing high-resolution imaging along focal axes FOV [127].

#### 3.3.3. Dynamically Controlling Terahertz Wavefronts with Cascaded Metasurfaces

**Figure 8:**
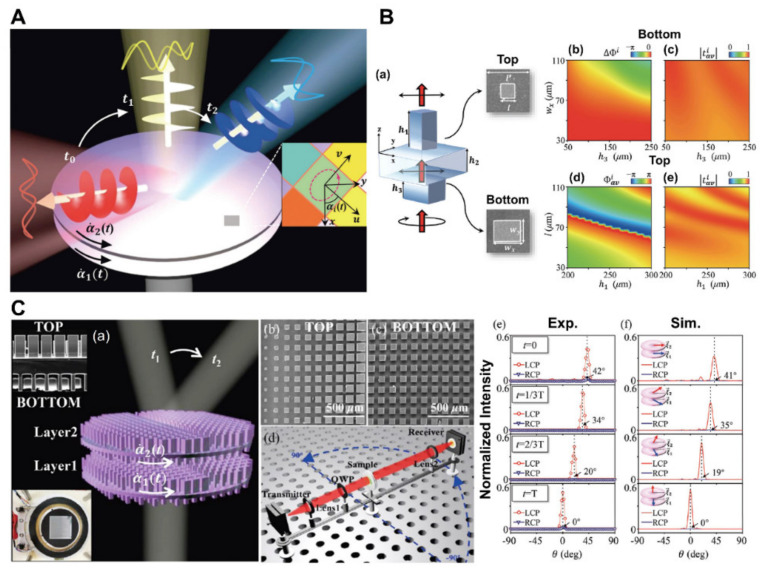
(**A**) Dynamic wavefront control using cascaded metasurfaces. (**a**) Diagram of the dynamic wavefront control process where the wave’s direction and polarization state change concurrently. The system is multi-layered (layer number) [128]. (**B**) (**a**) Illustrates a meta-atom composed of dual silicon (Si) posts. These are placed on opposing sides of the Si spacer. Because of its inhomogeneous structure, the remaining portions of the meta-atom can aid in accumulating sufficient transmission phases. FDTD simulated results, (**b**) transmission phase, (**c**) average transmission amplitude |t^i^_av_| against the parameters Wx and h_3_ of the bottom post. The computed average transmission and average transmission amplitude against l and h_1_ are illustrated in (**d**,**e**), receptively [128]. (**C**) The dynamic beam-steering metadevice’s findings. (**a**) A metadevice schematic with two layers of transmissive metasurfaces connected by a motorized rotation stage. Side-view SEM micrographs of a single metasurface with two layers are shown in the upper left inset. (**b**) Top and (**c**) bottom SEM micrographs of the manufactured metadevice. (**d**) A test configuration for characterizing the metadevice. (**e**,**f**) experimentally measured and simulated far-field scattering power distributions as the metadevice moves along Path I at different times. The metadevice travels along with Paths I and II, with solid lines (star signs) representing experimental results. The blue area represents a constant beam-steering angle [128]. (**A**–**C**) Reprinted/adapted with permission from Ref. [128]. Copyright 2021, SPIE and Chinese Laser Press.

The terahertz (THz) technology has recently attracted the attention of researchers due to its significant practical applications in high-speed communication systems, radar, and detectors [128]. Researchers have created a slew of cascaded metadevices for imaging, biomedical, and chemical sensing applications by controlling wavefronts in the terahertz region [129,130,131]. A cascaded metadevice, as shown in Figure 8A, can transform the impactful Jones-matrix property of the entire system by rotating different layers (each with a different phase profile) at different speeds, allowing exceptional manipulations of the wavefront and polarization features of a Terahertz beam imposing upon this device [128].

Each metadevice comprises two meticulously designed all-silicon transmissive metasurfaces with varying phases, as shown in Figure 8B. The first metadevice can effectively reroute a typical incoming Terahertz beam to detect across a wide solid-angle spectrum, whereas a second metadevice is used to change the wavefront and polarization of a terahertz beam in real time [128]. Dynamic beam steering and polarization control are achieved by combining two identical transmissive metasurfaces with different phase distributions. Figure 8C depicts a motorized stage that regulates the speed at which the two layers spin.

Additionally, two kinds of cascaded metasurfaces were also investigated to control the wavefronts of reflected and transmitted waves in the terahertz region. When the polarization of the incident field is parallel to the strip grating orientation, reflected and transmitted waves have identical amplitudes and a constant phase difference when the element size is altered. Based on the suggested element, the metasurface may deflect both transmitted and reflected wavefronts, but only by the same angle. By cascading four metallic layers, the independent amplitude and phase control of transmitting and reflecting waves can be achieved [132]. Compared with previously demonstrated metasurfaces in the terahertz region, the proposed components are compact, economical, and lighter. Therefore, they can be used as beam splitters in inexpensive THz imaging and detection systems. However, increasing the layers from two to four will make the fabrication costly and complex.

#### 3.3.4. Cascaded Metasurfaces Holography

Since the conventional single-layer metasurface provides a fixed degree of freedom owing to its reflection symmetry with reference to its structural axis, on the other hand, the multilayer cascaded metasurfaces’ components may be swapped, translated, or rotated. For example, cryptographic technologies are now widely used for security purposes such as intellectual property rights protection and product verification. The secret sharing technique, a well-known cryptographic primitive, was proposed by Adi Shamir and George Blakley to store sensitive information such as encryption keys for encoded data. They address the cryptographic problem of distributing a secret among several owners such that it can be recreated when a significant number of shares are available. In the optical implementation, information may be physically split up and reassembled using the cascaded holography approach. Here, we discuss recent advances in the multilayer cascaded metasurface layout for novel secret sharing and encryption platforms. Such methods can be developed to secure sensitive data against stealing and hacking. A common way to strengthen data security is to utilize information encryption.

**Figure 9:**
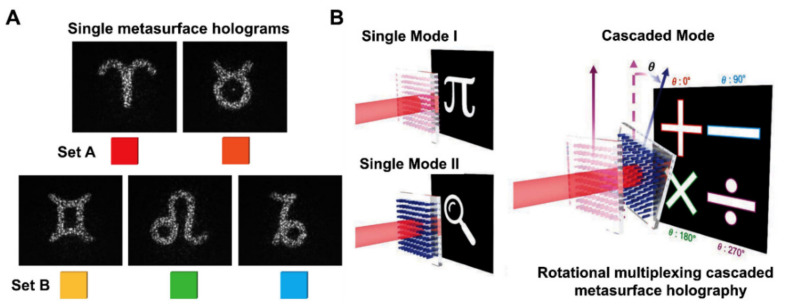
(**A**) Holographic encryption scheme. A “master share” metasurface hologram with three separate “deputy shares” is shown. On one substrate (set A), the researcher created two master share metasurface holograms and three deputy share metasurface holograms (set B). Fourier holograms for the astrological sign are encoded by a combination of metasurfaces of both sets and stacks. Each metasurface hologram has its own color for easier visualization. Measured cascaded metasurface holographic images are almost as good as their single-layer counterparts [133]. (**B**) The rotating multiplexing cascaded metasurfaces are seen schematically. Six different holographic pictures can be reconstructed using single-layer approaches I and II and the cascaded approach [134]. (**A**) Reprinted/adapted with permission from Ref. [133]. Copyright 2021, American Association for the Advancement of Science (AAAS). (**B**) Reprinted/adapted with permission from Ref. [134]. Copyright 2022 Wiley-VCH.

Based on metasurface holography, researchers provide an all-optical approach for secret information sharing. Recently a research group proposed a cascaded metasurface scheme for secret information sharing employing meta-surface holography. The metasurface holography is utilized as a spatially isolated share that transmits encoded data in the form of holograms. They merged by joining two of these shares. Light passing through a stack of metasurfaces collects hologram phase shifts and reconstructs the secret with high resolution. Furthermore, holograms formed from individual metasurfaces can be used to identify the shareholder clearly [133]. The schematic of the proposed scheme is shown in Figure 9A. Such as a study was carried out to investigate in-plane rotation as a unique multiplexing dimension and propose an efficient method for the cascaded metasurface holography. Six distinct holographic visualizations have been created and tested in only one cascaded approach. The two main modes of the proposed rotational multiplexing cascaded metasurfaces are conceptually represented in Figure 9B.

## 4. Generation of Vector Vortex Beams

**Figure 10:**
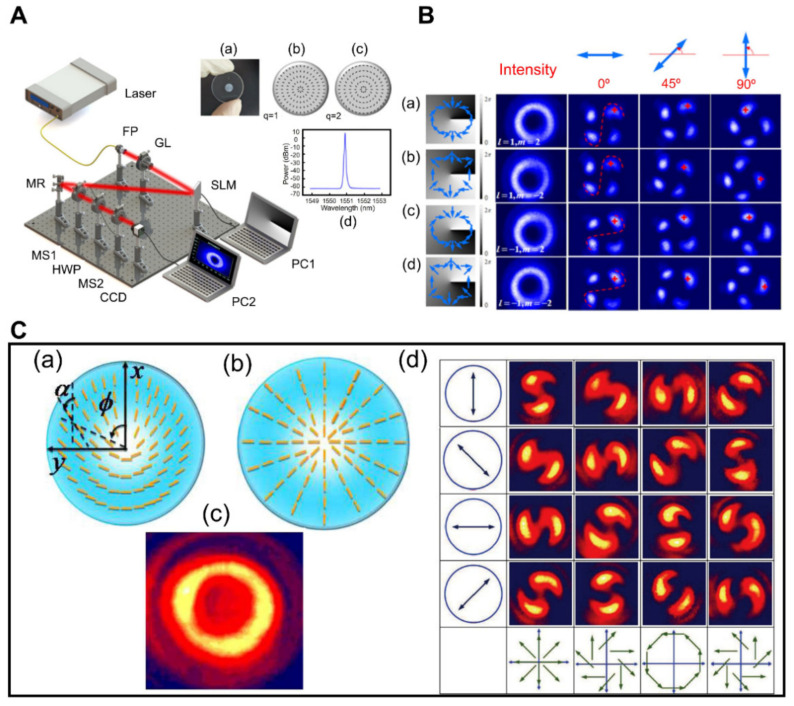
(**A**) Experimental setup for CVV beams (**right**). (**a**) Illustration of metasurface utilized in the experiment. (**b**,**c**) theorical estimated optical plane distribution of metasurface for q = 1 and q = 2 respectively. (**d**) The measured intensity distortion of the CVV beams with respect to wavelength [141]. (**B**) (**a**–**d**) display the measured intensity distribution of CCV beams with opposite sign of polarization orders (m) and charges (l). Polarization order is positive when the light spot’s rotation direction matches GL’s transmission axis rotation direction. As indicated by the red color dotted line, once two light spots on a similar diameter make an “s” shape, the charge is positive; when they form an anti “s” shape, the charge is negative (**left**) [141]. (**C**) Insets (**a**,**b**) show schematics of metasurfaces. A nanoscale waveplate with spatially changeable fast axis orientations is used to create nanograting based on variable space. Inset: (**c**) The intensity profile of the CVV beams shows doughnut characteristics. (**d**) CVV beams are created experimentally when GLP1 is positioned at 45 degrees. The polarization direction of the Glan laser polarizer (GLP3) is displayed in the first column line. Polarization angles from polarization analyzers are given in the four columns of data below, and the lower rows show rebuilt vector fields for the output beams [142]. (**A**,**B**) Reprinted/adapted with permission from Ref. [141]. Copyright 2017 IEEE. (**C**) Reprinted/adapted with permission from Ref. [142]. Copyright 2014 Optica Publishing Group (OPG).

With its spiral phase front and spatially anisotropy polarization, the cylindrical vector vortex CVV beam has grasped much attention due to better stability and beam integrity. Moreover, it also provides a new degree of freedom and can be employed in a variety of applications, including communication systems [135,136,137,138], beam manipulation, and optical imaging [139,140]. Recently a novel method of generation CVV beam has been explored using a q-plate scheme and a programmable SLM. The q-plate scheme consists of cascaded dual metasurfaces and a half-wave plate that can adjust the CVV beam’s polarization order up to eight, as shown in Figure 10A,B [141]. Additionally, the researchers have come up with a simple and effective way to generate CVV beams using two cascaded metasurfaces, as illustrated in Figure 10C. The metasurface works as a PB phase part that changes in space and can make vortex phase and vector polarization. One of the cascaded metasurfaces is used to change the sign of topological charges linked with vortices, and the other metasurface is used to change local polarization. However, the formations of vortex beams in the broadband millimeter spectral range were not investigated.

**Figure 11:**
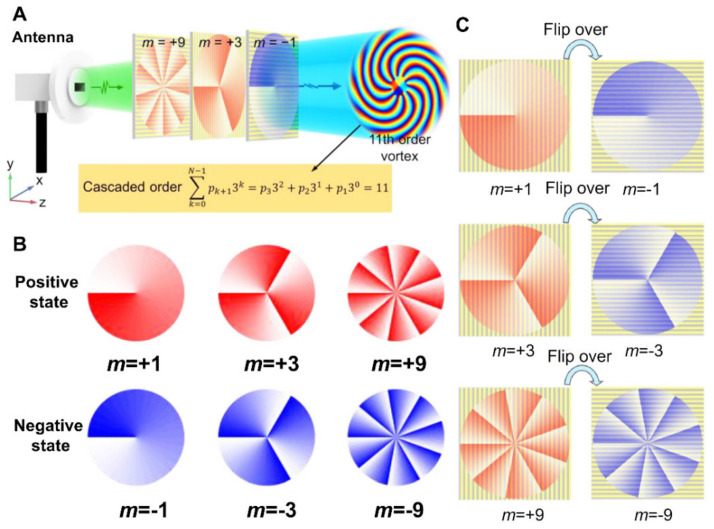
(**A**) Schematic of mechanism to produce 11th order vortex beam generation (VBG) by cascading three basic vortex generation of −1, −3, −9 order, respectively [143]. (**B**) Illustration of transverse phase distribution. (**C**) Illustrations demonstrate the formation of the inverse order vortex by flipping over the vortex beam [143]. (**A**–**C**) Reprinted/adapted with permission from Ref. [143]. Copyright 2022, De Gruyter.

Recently, the researchers demonstrated the generation of a vortex beam with a large topological number by cascading three metasurfaces, as demonstrated in Figure 11. The reconfigured vortex beam generation is stimulated by the idea of the base-3 concept [143].

## 5. Conclusions and Outlook

In this review paper, we discussed in detail the metasurface from fundamental concepts and theory to their potential application. Metasurfaces have become a trending research topic in the past few years because of their great efficiency and unique functionalities, which were not achievable with traditional bulky optical elements. Moreover, the ease and low cost of the fabrication process is compatible with existing systems, sparking their importance in the semiconductor industry. Additionally, the metasurface offers appealing features at the subwavelength, including manipulating phase, polarization, amplitude, and other exotic properties of light, making the metasurface superior to other traditional diffractive optical elements. Furthermore, programmable and controllable metasurfaces can be generated by employing innovative materials with dynamic optical characteristics.

We anticipate that metasurfaces will eventually make their way into industry when the appropriate application fulfills the requirements for commercial manufacturing. However, we do not expect that they will take the place of traditional diffractive and refractive optics since conventional diffractive lenses are sufficient and more economical for numerous applications. Up to date, we have already seen exciting advancements in the field of the optical metasurface. Still, more efforts are still required to develop its application in various fields. For example, in many flat devices, the metals serve as a perfect electric conductor at low frequencies, but, at the optical frequency regime, the ohmic losses of metals become significant and suffer the performance of a metasurface. The utilization of high-k dielectric materials might be a promising choice for metasurfaces operating at optical frequencies.

In addition, in the passive operation, the functionalities of metasurfaces have been extended by developing a multilayer stack, which causes losses and creates many nanofabrication challenges. Although passive metasurfaces have numerous potential applications, it is still preferable to control and tune their optical properties under external stimuli. Various approaches have been utilized for this purpose. However, still, it is challenging to achieve a tunable metasurface with a large range of phase/amplitude tuning, a fast switching speed, and lower power consumption. Therefore, looking for novel materials with intriguing tunable optical properties are needed.

From a fabrication point of view, metasurface fabrication technologies should be consistent with present low-cost, large-scale semiconductor technologies. However, this may already be conceivable for devices operating in the near- and mid-IR (above 1.5 μm wavelengths). However, it is more challenging for devices working below 1 μm, which are almost entirely manufactured through electron beam lithography. In theory, large-scale manufacturing methods such as deep UV lithography, roll-to-roll (R2R) nanoimprint lithography, and soft lithography might handle this difficulty, but practical constraints must be addressed before this becomes a reality.

In this review, we sum up imagining some of the most relevant metasurfaces research fields, also with the intention of offering strategic direction to other scholars.

- Exploring and developing new materials with innovative characteristics such as metasurface ingredients will be a future research focus. In the last decade, the advent of novel materials such as graphene, ITO, VO_2_, and TiO_2_ has sparked several optical applications. These materials, with their distinct optoelectronics properties, demonstrate novel metasurface functions. Furthermore, materials that can overwhelm the significant loss of plasmonic materials while retaining the intriguing properties of plasmonic resonances are in high demand for metasurfaces.
- Metasurfaces featuring wideband responses and controllability are another field to examine. Metasurfaces are being used to achieve several functionalities of classic optics. Furthermore, the narrowband and considerable spectrum scattering of metasurfaces are significant drawbacks. Several optical parts, e.g., lenses, need wide bandwidth as well as minor spectrum aberration. Furthermore, metasurfaces ought to be able to modify their features flexibly in order to be used in devices, including displays and light modulators. The design and implementation of a fully reconfigurable and programmable metasurface is in high demand.
- The third area of research is the implementation of metasurfaces in current systems could be the quickest approach to getting metasurface innovation to the market. This notion possesses the potential to be employed in numerous applications, including clinical surveillance, imaging, optical communication, optical laser beams, and thermo-detectors.
- Designing metasurfaces with promising properties and functionalities based on advanced machine learning computational techniques is another novel design approach. There is a great demand to explore these advanced machine learning approaches in optics and photonics, where metasurfaces play an essential role.

## Data Availability

Data sharing not applicable as no new data were created or analyzed in this paper.
